# Use of Radiotherapy in the Treatment of Non-melanoma Skin Cancer: Experience From a Regional Referral Center

**DOI:** 10.7759/cureus.107122

**Published:** 2026-04-15

**Authors:** Oliver A Colis Arenas, Katya C Navarro Montellano, Cruz Alejandro Valdivia Esquivel

**Affiliations:** 1 Research, Centenario Hospital Miguel Hidalgo, Aguascalientes, MEX; 2 Medicine, Universidad Autónoma de Aguascalientes, Aguascalientes, MEX; 3 Oncology/Radiotherapy, Centenario Hospital Miguel Hidalgo, Aguascalientes, MEX

**Keywords:** case series, local failure, non-melanoma skin cancer, radiotherapy, treatment toxicity

## Abstract

Background

Non-melanoma skin cancer is the most common malignancy worldwide. Although surgery remains the treatment of choice in most cases, radiotherapy represents an important alternative in patients who are not candidates for resection, in anatomically complex locations, or as adjuvant therapy. Although radiotherapy has been associated with favorable local control rates in multiple studies, evidence from real-world clinical practice, particularly in heterogeneous cohorts and locally advanced disease, remains limited. The aim of this study was to describe the clinicopathological characteristics, radiotherapy schemes, local outcomes, and toxicity in patients treated at a regional referral center.

Methods

A retrospective descriptive case series was conducted at a regional referral hospital in Mexico. Patients with histopathologically confirmed non-melanoma skin cancer treated with radiotherapy during 2023 were included. Demographic, tumor-related, treatment, and follow-up variables were analyzed using descriptive statistics. Clinical outcomes, including local failure and toxicity, were assessed based on retrospective clinical documentation without standardized response criteria or validated toxicity scales.

Results

A total of 18 patients were included, with a mean age of 74.2 years; 66.7% were male. The most frequent histologies were basal cell carcinoma (44.4%) and squamous cell carcinoma (38.9%). Two-thirds of cases corresponded to locally advanced disease (T3-T4). The series was heterogeneous regarding treatment intent and radiotherapy modality. The mean administered dose was 49 Gy in 17 fractions. During a mean follow-up of 7.7 months, 15 patients (83.3%) had no documented local failure during the available follow-up period, while three patients (16.7%) did. Late toxicity was infrequently reported and mainly limited to cutaneous changes, based on retrospective clinical documentation. These findings reflect the clinical course observed during the available follow-up period and should be interpreted within the context of its duration and the retrospective, non-standardized nature of outcome assessment.

Conclusion

Findings from this case series should be interpreted strictly as descriptive observations derived from routine clinical practice, given the small sample size, clinical heterogeneity, lack of standardized outcome assessment, and limited follow-up duration.

## Introduction

Non-melanoma skin cancer is the most common malignancy worldwide, with increasing incidence, particularly among older populations and in sun-exposed areas of the head and neck [1,2]. Basal cell carcinoma and squamous cell carcinoma account for the majority of cases, while other subtypes are less common [1,2]. Major risk factors include chronic ultraviolet radiation exposure, advanced age, and immunosuppression [1].

Surgery remains the treatment of choice in most cases; however, radiotherapy plays an important role as definitive treatment in patients who are not surgical candidates, in lesions located in areas where resection may lead to significant functional or cosmetic sequelae, and as adjuvant therapy in the presence of high-risk features [1-4]. Common indications for postoperative radiotherapy include unresectable positive margins, perineural invasion, large tumors, recurrence, and aggressive histological features [1-3].

Radiotherapy can be delivered using different modalities, including superficial radiotherapy, electrons, photons, or brachytherapy. The choice depends on tumor depth, anatomical location, available technology, and treatment intent, leading to variability in clinical practice [1,2,4]. In elderly patients, hypofractionated regimens have gained relevance due to their practicality and good tolerability, with favorable local control outcomes reported in several series [3,5-7].

Despite this, much of the available evidence comes from retrospective studies with heterogeneous populations and variability in treatment techniques and regimens [2-5]. Although favorable local control rates with radiotherapy have been reported, these findings are primarily derived from early-stage and more homogeneous cohorts. Additionally, institutional reports from real-world settings in Mexico and Latin America are limited, particularly in elderly populations and locally advanced disease.

Therefore, the aim of this study was to describe the clinicopathological characteristics, radiotherapy schemes, local outcomes, and observed toxicity in patients with non-melanoma skin cancer treated at a regional referral center.

## Materials and methods

Study design

A retrospective, observational, descriptive, single-center case series was conducted. Given the retrospective nature of the study, no standardized criteria for tumor response or toxicity grading were systematically applied. Therefore, all reported outcomes should be interpreted as descriptive observations derived from routine clinical documentation.

Study setting and period

Patients with non-melanoma skin cancer treated with radiotherapy at the Centenario Hospital Miguel Hidalgo between January 1, 2023, and December 31, 2023.

Study population

Consecutive patients with histopathologically confirmed non-melanoma skin cancer who received radiotherapy and had available clinical information in electronic medical records were included. Each patient had a single treated lesion, which was considered the unit of analysis.

Patients with incomplete records that prevented evaluation of main variables or without post-treatment clinical follow-up were excluded.

Data source

Data were obtained through review of institutional electronic medical records.

Variables and operational definitions

The variables included in the study, along with their operational definitions and measurement methods, are described in Table 1.

**Table 1:** Variables and operational definitions

Variable	Type	Operational definition	Categories / Measurement
Age (years)	Continuous	Age at the start of radiotherapy	Mean
Sex	Dichotomous	Recorded biological sex	Male/Female
Immunosuppression	Dichotomous	Condition associated with immunosuppression	Yes/No
Histology	Nominal	Histopathological diagnosis	Basal cell/Squamous cell/Others
Tumor size (cm)	Continuous	Maximum tumor diameter	Mean
T stage	Ordinal	Clinical classification	T1–T4
Anatomical site	Nominal	Tumor location	Head and neck/Others
RT modality	Nominal	Type of radiotherapy	Superficial/Electrons/Photons
Treatment intent	Nominal	Therapeutic objective	Curative/Adjuvant/Palliative
Total dose (Gy)	Continuous	Total administered dose	Mean
Fractions (n)	Discrete	Number of treatment sessions	Mean
Dose per fraction (Gy)	Continuous	Dose per session	Mean
Prior surgery	Dichotomous	Previous surgical treatment	Yes/No
Margins	Nominal	Post-surgical status	Negative/Positive/Unknown/NA
Local failure	Dichotomous	Clinical persistence or recurrence	Yes/No
Late toxicity	Nominal	Recorded cutaneous events	None/Mild/Moderate - Severe
Follow-up (months)	Continuous	Time to last evaluation	Mean

Outcome definition

Local failure was defined as persistence of tumor after treatment or reappearance of the lesion at the treated site during follow-up, based on clinical assessment documented by the treating physician. Given the retrospective nature of the study, it was not possible to consistently distinguish between persistent disease and recurrence after initial response, nor to obtain systematic imaging or histopathological confirmation. Accordingly, this variable should be interpreted as a pragmatic clinical descriptor rather than a standardized oncological endpoint.

Follow-up

Follow-up time was defined as the interval between completion of radiotherapy and the last available clinical evaluation. Mean follow-up was 7.7 months.

Toxicity

Late toxicity was defined as cutaneous adverse events documented in follow-up notes after treatment completion. Due to the retrospective design, neither a standardized assessment time point nor a validated toxicity scale (e.g., CTCAE) could be consistently applied. Therefore, toxicity findings are likely underestimated and should be interpreted in a descriptive context.

Statistical analysis

A descriptive analysis was performed. Categorical variables were expressed as frequencies and percentages, while continuous variables were summarized as means. No inferential or time-to-event analysis was performed due to the descriptive nature of the study, limited sample size, and lack of standardized outcome assessment.

Ethical considerations

This study was conducted through retrospective review of electronic medical records, without direct patient intervention. No identifiable data were collected, and confidentiality was ensured through data anonymization. According to institutional regulations and ethical review, individual informed consent was not required.

## Results

**Figure 1:**
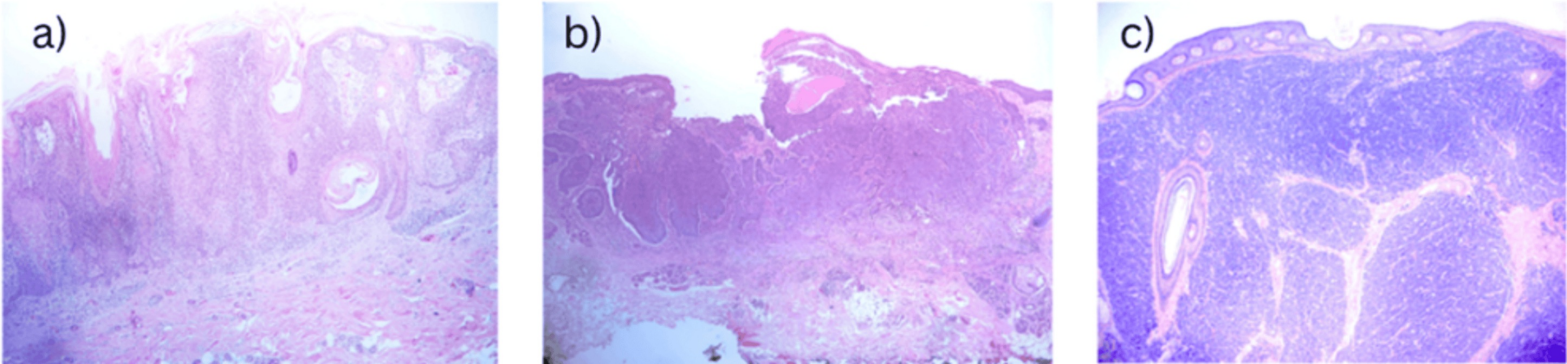
Representative histopathological findings of non-melanoma skin cancer. (A) Moderately differentiated squamous cell carcinoma with keratinization, showing invasive growth into the dermis. (B) Basal cell carcinoma with an infiltrative pattern. (C) Poorly differentiated carcinoma, morphologically consistent with Merkel cell carcinoma. Hematoxylin and eosin (H&E) staining; original magnification ×10

A total of 18 patients with non-melanoma skin cancer treated with radiotherapy were included. Clinical, tumor, and treatment characteristics are summarized in Table 2. The population mainly consisted of older adults, with a slight male predominance. The most frequent histologies were basal cell carcinoma and squamous cell carcinoma (Figure 1). Notably, 12 of 18 patients (66.7%) had locally advanced disease (T3-T4).

**Table 2:** Clinical, pathological and treatment characteristics of the case series

Variable	Category	n (%) or mean
Total number of patients	—	18
Age (years)	—	74.2
Sex	Male	12 (66.7%)
	Female	6 (33.3%)
Immunosuppression	No	17 (94.4%)
	Yes	1 (5.6%)
Histology	Basal cell	8 (44.4%)
	Squamous cell	7 (38.9%)
	Merkel	1 (5.6%)
	Basosquamous	1 (5.6%)
	Mixed	1 (5.6%)
Tumor size (cm)	—	3.9
T stage	T1	2 (11.1%)
	T2	4 (22.2%)
	T3	6 (33.3%)
	T4	6 (33.3%)
Anatomical site	Nose	5 (27.8%)
	Cheek	3 (16.7%)
	Scalp	3 (16.7%)
	Forehead	1 (5.6%)
	Ear	1 (5.6%)
	Others	5 (27.8%)
Radiotherapy modality	Superficial kV	8 (44.4%)
	Electrons	5 (27.8%)
	Photons MV	2 (11.1%)
	Not specified	3 (16.7%)
Treatment intent	Curative	11 (61.1%)
	Adjuvant	2 (11.1%)
	Palliative	1 (5.6%)
	Not specified	4 (22.2%)
Total dose (Gy)	—	49 (range 35 - 66)
Number of fractions	—	17 (range 5- 33)
Dose per fraction (Gy)	—	3.3
Prior surgery	Yes	9 (50.0%)
	No	9 (50.0%)
Surgical margins	Negative	3 (16.7%)
	Positive	0 (0.0%)
	Unknown	6 (33.3%)
	Not applicable	9 (50.0%)
Follow-up (months)	—	7.7
Local failure	No	15 (83.3%)
	Yes	3 (16.7%)
Late toxicity	None	13 (72.2%)
	Telangiectasia + fibrosis	2 (11.1%)
	Telangiectasia + atrophy	2 (11.1%)
	Ulcer	1 (5.6%)

There was variability in treatment intent and radiotherapy modalities. Radiotherapy was mainly used with curative intent, although adjuvant and palliative cases were also included; in some patients, this information was not documented. The most frequent modality was superficial radiotherapy, followed by electrons. Treatment regimens were also variable, with a mean dose of 49 Gy in 17 fractions.

During a mean follow-up of 7.7 months, three patients (16.7%) developed clinically documented local failure, while no such events were recorded in the remaining patients.

Patients with local failure represented a small proportion of the cohort and were heterogeneous in terms of tumor stage, lesion size, radiotherapy modality, and treatment intent. Individual characteristics of these cases are shown in Table 3.

**Table 3:** Clinical characteristics of patients with local failure (n=3, 16.7% of the cohort)

Variable	Case 1	Case 2	Case 3
Age (years)	68	78	85
Sex	Female	Male	Female
Histology	Basal cell	Squamous cell	Squamous cell
Site	Nose	Cheek	Ear
T stage	T2	T2	T3
Tumor size	3.5	2.8	4.2
Prior surgery	No	Yes	No
Margins	NA	Unknown	NA
Dose (Gy)	55	50	55
Intent	Curative	Curative	Curative

Patients with local failure represented a small proportion of the cohort and were heterogeneous in terms of tumor stage, lesion size, radiotherapy modality, and treatment intent. Among these cases, patients were older adults (range 68-80 years), and all were treated with curative intent. Two cases corresponded to squamous cell carcinoma and one to basal cell carcinoma. Tumor stage ranged from T2 to T3, with the only T3 case being a squamous cell carcinoma located in the ear. Tumor size ranged from 2.8 to 4.2 cm.

Only one patient had a history of prior surgical resection, with no available information regarding margin status; the remaining two patients had not undergone prior surgery. All patients received total doses between 50 and 55 Gy.

In a descriptive comparison, local failure was observed in patients with larger tumors and more advanced stages; however, no formal statistical analysis was performed, and these observations should be interpreted cautiously given the small number of events.

Regarding late toxicity, adverse events were infrequently reported and mainly consisted of cutaneous changes. Assessment was based on clinical documentation in follow-up notes.

## Discussion

In this case series, patients treated with radiotherapy for non-melanoma skin cancer were predominantly older adults, with male predominance and basal and squamous histologies, consistent with previous reports [1-8].

A relevant aspect of this series is the high proportion of locally advanced disease (66.7%). This clinical profile differs from many published series, which often include earlier-stage disease or more homogeneous populations. In this context, the present study illustrates the use of radiotherapy in real-world clinical practice at a public referral center, rather than aiming to establish direct comparisons of efficacy. Accordingly, the study was not designed to evaluate treatment effectiveness, and any interpretation beyond descriptive reporting should be avoided.

During the available follow-up, most patients did not have documented local failure. However, this finding should not be interpreted as equivalent to local control, given the short follow-up and lack of standardized assessment. Published series have reported high local control rates with radiotherapy, frequently exceeding 85-95% in early-stage non-melanoma skin cancer and in more homogeneous cohorts treated with standardized approaches [3,5,6,8-10]. However, direct comparison with the present series is limited due to differences in stage distribution, treatment heterogeneity, and follow-up duration. In contrast, the present series included a heterogeneous population with different histologies, treatment intents, and a high proportion of locally advanced disease, which limits direct comparison with those benchmarks. Additionally, outcomes were based on clinical documentation without systematic imaging or histopathological confirmation, which affects the interpretability of local failure as a clinical endpoint and precludes differentiation between persistence and true recurrence.

Cases with local failure were few and heterogeneous, precluding identification of clear patterns or associations. Only one patient had prior surgery, with no information on margin status, further limiting interpretation.

Although the sample size precludes formal statistical analysis, descriptive observations were made. No consistent patterns could be established; local failure occurred in patients with larger tumors and more advanced stages, but these findings should be interpreted cautiously, given the small number of events. Additionally, two of the three cases corresponded to squamous cell carcinoma, which may reflect its known more aggressive biological behavior compared to basal cell carcinoma. However, these observations are purely descriptive and do not allow for any inference of association.

Radiotherapy was used in different settings (curative, adjuvant, and palliative) and with various modalities, likely reflecting individualized decision-making based on patient characteristics, tumor features, and resource availability. The average dose corresponded to a moderately hypofractionated regimen, consistent with reports in elderly populations where balancing efficacy and tolerability is essential [3,5,7].

The mean administered dose (49 Gy in 17 fractions) is broadly consistent with moderately hypofractionated regimens commonly used in elderly patients with non-melanoma skin cancer, where treatment convenience and tolerability are important considerations [3,5,7]. Nevertheless, given the heterogeneity in tumor stage, histology, treatment intent, and radiotherapy modality, this study was not designed to assess dose-response relationships or to determine the adequacy of any specific regimen in relation to local outcomes. It was not possible to assess whether delivered doses were optimal according to current radiotherapy standards.

Regarding toxicity, most patients did not present relevant late events, and those recorded were mainly cutaneous. However, due to the retrospective design and lack of standardized scales, underreporting, particularly of mild or cosmetic effects, is likely.

This study has several important limitations. It is a retrospective, single-center case series with a small sample size, limiting generalizability. There is also heterogeneity in multiple clinical and treatment variables, as well as missing relevant data, including treatment intent and surgical margin status. An additional limitation relates to the lack of detailed radiotherapy technical parameters. Due to the retrospective design and variability in clinical documentation, information regarding target volumes, treatment margins, field design, use of bolus, and dose constraints was not consistently available. This limits the ability to fully characterize treatment delivery and compare technical aspects with standardized protocols. Finally, lack of standardized response and toxicity assessment, along with short follow-up, limits the interpretation of outcomes. No time-to-event analysis or survival estimation was feasible due to the short and heterogeneous follow-up.

Despite these limitations, this series provides insight into the use of radiotherapy in a real-world oncological setting in Mexico. It describes an elderly population with a high proportion of locally advanced disease treated with hypofractionated regimens under routine clinical conditions. These findings help contextualize radiotherapy use in local practice, support clinical decision-making, and generate hypotheses for future prospective studies with longer follow-up.

## Conclusions

This case series describes the use of radiotherapy in patients with non-melanoma skin cancer in a real-world clinical setting, characterized by an elderly population with a high proportion of locally advanced disease and heterogeneous treatment approaches.

The findings should be interpreted strictly as descriptive observations, given the small sample size, clinical heterogeneity, lack of standardized outcome assessment, and limited follow-up duration.
